# Natural sonic crystal absorber constituted of seagrass (Posidonia Oceanica) fibrous spheres

**DOI:** 10.1038/s41598-020-79982-9

**Published:** 2021-01-12

**Authors:** L. Barguet, V. Romero-García, N. Jiménez, L. M. Garcia-Raffi, V. J. Sánchez-Morcillo, J.-P. Groby

**Affiliations:** 1 Laboratoire d’Acoustique de l’Université du Mans (LAUM), UMR CNRS 6613, Institut d’Acoustique - Graduate School (IA-GS), CNRS, Le Mans Université, Le Mans, France; 2 Instituto de Instrumentación para Imagen Molecular, Consejo Superior de Investigaciones Científicas, Universitat Politècnica de València, Camino de vera s/n, 46022 Valencia, Spain; 3 Instituto Universitario de Matemática Pura y Aplicada (IUMPA), Universitat Politècnica de València, Camino de vera s/n, 46022 Valencia, Spain; 4 Instituto de Investigación para la Gestión Integrada de Zonas Costeras, Universitat Politècnica de València, Paranimf, 46730 Gandia, Spain

**Keywords:** Acoustics, Bioinspired materials

## Abstract

We present a 3-dimensional fully natural sonic crystal composed of spherical aggregates of fibers (called Aegagropilae) resulting from the decomposition of Posidonia Oceanica. The fiber network is first acoustically characterized, providing insights on this natural fiber entanglement due to turbulent flow. The Aegagropilae are then arranged on a principal cubic lattice. The band diagram and topology of this structure are analyzed, notably via Argand representation of its scattering elements. This fully natural sonic crystal exhibits excellent sound absorbing properties and thus represents a sustainable alternative that could outperform conventional acoustic materials.

## Introduction

**Figure 1:**
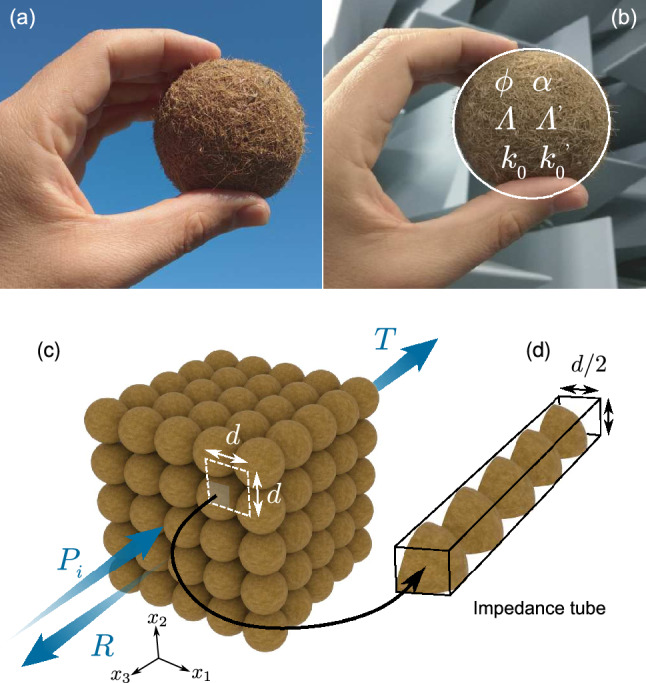
Aegagropilae (**a**) and its model as a porous sphere (**b**). (**c**) Principal cubic arragement of Aegagropilae used to construct the fully natural 3D sonic crystal. (**d**) Reduced model used for the scattering measurement in the impedance tube at normal incidence.

Complex biological structures usually result from the adaption of living bodies to their environmental constraints. These structures generally ensure several functionalities in nature^1^. Very few of them are the result of natural dynamic processes involving organic waste, apparently formed for no particular reason and without ensuring any specific functionality. Aegagropilae such as Posidonia balls are the archetype of these organic structures. They are natural spherical aggregates formed by entangled fibers as a result of the decomposition of Posidonia Oceanica, as shown in Fig. 1a. This marine plant is endemic of the Mediterranean sea and forms large underwater meadows that are of particular importance for the marine ecosystem. Aegagropilae can be easily collected on Mediterranean sea cost. Since the pioneer work of Cannon^2^, which first showed the formation of a Aegagropilae from fibers in a washing machine tray and thus attributed the entanglement process of the Posidonia leaves to the turbulent flow created by the sea motion, the fully natural formation process of Aegagropilae has been widely investigated^3, 4^. The study of Aegagropilae fiber network is indeed relevant for green manufacturing process of paper, felt or non-woven textile. Recently, Verhille *et al.*^5^ indirectly studied this formation process by assessing the structure and mechanical properties of a large number of hand-collected Aegagropilae. Acoustic characterization also appears as a complementary and efficient tool to assess the fibrous network structure and possibly the entanglement process of Aegagropilae fibrous network. Currently, Aegagropilae fibers are used to produce sustainable nano-composite^6^, but no direct application has been found yet to fully natural Aegagropilae.

While photonic structures have been proven to exist in several natural systems^7, 8^, most of the sustainable acoustic or elastic metamaterials are either man modified^9^ or bio-inspired^10, 11^. Very recently^12^, the first natural ultrasonic metamaterial was reported. Some moth species evolve wings covered with scales that reduce ultrasonic echoes to avoid bat predation. The structure, efficient in the ultrasonic regime, cops with only one of the two (symmetric and antisymmetric) problems necessary to achieve perfect absorption in transmission problem, thus reaching 0.7 absorption. We therefore propose a fully natural organic sonic crystal composed of Aegagropilae, taking advantages of their almost spherical geometries, as a complement in the audible frequency range to the first natural acoustic structured materials.

Phononic or sonic crystals are periodic arrangements of scatterers allowing the control of acoustic or elastic waves in an unprecedented way. A number of applications of sonic crystals have been proposed, such as acoustic filters^13, 14^ waveguides^15^, wave traps^16^, lenses^17, 18^, sound diffusers^19^, and acoustic barriers for traffic noise attenuation^20^. Most sonic crystals are thus composed of rigid scatterers embedded in air. Phononic crystals constituted of poroelastic spheres have been lately studied numerically when immersed and saturated by water and their insulation and absorption efficiencies were estimated^21^. Nevertheless, the contributions of the viscothermal losses of the wave which propagates mainly in the fluid phase and of the viscoelasticity of the poroelastic skeleton to the global system dissipation have not been analyzed independently of each other. The proposed fully natural organic sonic crystal is constituted of dissipative and soft-porous spheres, the fiber network micro-structure of which is assessed in this article via acoustic characterization, see Fig. 1b. The spherical Aegagropilae are periodically arranged on a principal cubic lattice in air, as shown in Fig. 1c. The skeleton is also considered motionless, thus allowing to only account for the slow wave, i.e., the Aegagropilae fiber network is modeled as an equivalent fluid and only waves propagate in the fluid phase. In this way, only the effect of viscothermal losses are studied.

The present article addresses several issues. First, we use acoustic methods to characterize the Aegagropilae fiber network and assess its micro-structure. Second, a fully natural dissipative and soft sonic crystal constituted of Aegagropilae arranged over a principal cubic system is analyzed both experimentally and numerically in terms of band gaps translation. Third, the acoustic behavior of this fully natural sonic crystal is analyzed via the Argand diagram of both the reflection and transmission coefficients, enabling a novel way to explore the structure topology and the absorption efficiency. Aegagropilae fiber network sonic crystals are shown to be fully natural and highly efficient sound absorbing structures and thus sustainable alternatives that could overcome conventional acoustic materials.

## Results

Aegagropilae were collected in the mediterranean coastal village of Daimús (Spain). Nearly 50 almost spherical samples were cautiously collected in the morning and by hands to avoid any bias caused by human activities. They were selected after preparation based on their diameter and split into two categories: 15 samples were used for the fiber network acoustic characterization and 15 samples were considered to realize the 2-dimensional man modified sonic crystal (see Supplementary information) and the fully natural 3-dimensional sonic crystal.

### Acoustic characterization of the Aegagropilae fiber network and micro-structure assessment

**Figure 2:**
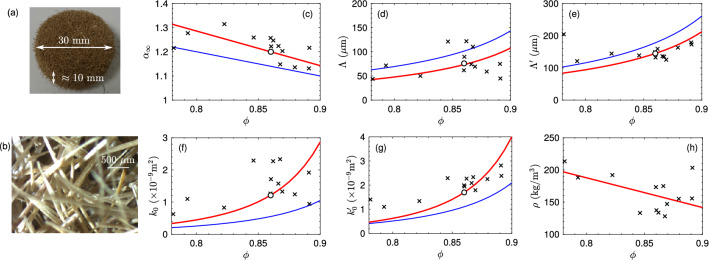
A picture of a sample is presented on (**a**), while a stereo microscopic image of the Aegagropilae fiber network is shown on (**b**). The tortuosity $\alpha _\infty$ (**c**), viscous $\Lambda$ (**d**) and thermal $\Lambda '$ (**e**) characteristic lengths and viscous $k_0$ (**f**) and thermal $k_0'$ (**g**) permitivities are shown in function of the porosity $\phi$ of the Aegagropilae fiber network. The markers are the experimentally recovered parameters, the dependency of each parameters are depicted in blue curves for the periodically aligned fiber model and in red curves for the homothetic transformation one. Open circles refer to the parameters that are used for the sonic crystal simulations. The density as a function of $\phi$ is depicted on (**h**) together with the linear approximation.

Cylindrical samples of $30\text { mm}$ in diameter, the thickness of which ranging from 12 to $14\text { mm}$, e.g. as depicted in Fig. 2a, were cut from the core of the first 15 Aegagropilae (see Methods). The outward shell built on a compaction process, where weak orthoradial excess in fiber orientation was noticed^5^, is thus not considered here. These 15 samples were acoustically characterized following the procedure described in Ref.^22^ in a 4 microphone circular cross-sectional impedance tube (see also Methods) in both the direct and reverse orientations to ensure their quasi-homogeneity (symmetric and reciprocal samples). The approximated thickness of $\approx 10\text { mm}$, was determined empirically to ensure the symmetry of the sample, i.e., identical reflections for both orientations, thus ensuring the homogeneity of the sample. Two samples were found inhomogeneous because of the presence of a inner macro-scale heterogeneity and were rejected. The 13 remaining samples were weighed via an analytical balance in order to determine their densities. The acoustic behavior of the Aegagropilae fiber network is assumed to be well described by the Johnson–Champoux–Allard–Lafarge model^23, 24^ (see Methods), which is commonly used for fibrous material. These samples have thus been considered as equivalent fluids with a complex and frequency dependent density and bulk modulus. They are described by the porosity $\phi$, the tortuosity, $\alpha _{\infty }$ which provides information on the fiber entanglement, the viscous and thermal characteristic lengths $\Lambda$ and $\Lambda '$ which provide averaged values of respectively the minimum and maximal distances bewteen the fibers within the network, and viscous and thermal permeabilities $k_0$ and $k_0'$. Figure 2c–g depicts the reconstructed parameters in function of the reconstructed porosity $\phi$ for the 13 samples. The measured density as a function of $\phi$ is also depicted in Fig. 2h and is found in good correlation with the porosity.

As a first approximation, the samples were assumed to be constituted of infinitely long fibers perpendicular to the sample height and arranged on a square lattice. Under this assumption, analytic formulae of the 5 parameters $\mathbf {q}=<\alpha _\infty , \Lambda , \Lambda ', k_0, k_0'>$ are provided for $\phi >0.6$ in Ref.^25^. These expressions depend on the fiber radius *a* and porosity $\phi$ which equals $1-\pi a^2/l^2$, where *l* is the lattice size of the fiber arrangement. Minimizing in the least square sense the difference between the measured parameters $\mathbf {q}^{meas}$ and $\mathbf {q}(a,l)$ provides $l\approx 130~\mu \text {m}$ and $a=29~\mu \text {m}$ which are qualitatively in accordance with the stereo microsopic (Olympus SZ61) image of the fibers shown in Fig. 2b. The corresponding results are plotted (blue curves) in Fig. 2c–g. Nonetheless, it is clear, notably from Fig. 2c, that the acoustic parameters of the Aegagropilae fiber network do not follow this simple model thus implying a more complex arrangement of the fibers.

Therefore, we propose another model, based on the homothetic transformation assuming the solid volume over the Representative Elementary Volume (REV) of volume *V* is constant. Introducing a compression/dilation rate $\eta =V^i/V=(1-\phi )/(1-\phi ^i)$ from an initial state, indicated by the exponent *i*, to another one and referring to Ref.^26^, the tortuosity reads as $\alpha _\infty =1-\eta (1-\alpha _\infty ^i)$. Referring to the definition of the thermal characteristic length, which is a purely geometric parameter and assuming that the pore surface is constant over the transformation, $\Lambda '=\phi /(\phi ^i \eta )\Lambda '^i$. A similar trend is inferred for the viscous characteristic length thus providing $\Lambda =\phi /(\phi ^i \eta )\Lambda ^i$. As far as it concerns the viscous permeability, Carman-Kozeny^27^ proposes $k_0=\zeta a_H^2 \phi /\alpha _\infty$, where $\zeta$ is a shape factor assumed constant during the transformation and $a_H$ is the hydraulic radius which correspond to $\Lambda '$. Therefore, we propose $k_0=(\Lambda '/\Lambda '^i)^2\phi \alpha _\infty ^i/(\phi ^i \alpha _\infty )k_0^i$ and a similar trend is inferred for the thermal permeability thus providing $k_0'=(\Lambda '/\Lambda '^i)^2\phi \alpha _\infty ^i/(\phi ^i \alpha _\infty )k_0'^i$. Again, minimizing in the least square sense the difference between the measured parameters $\mathbf {q}^{meas}$ and $\mathbf {q}(\phi ^i,\alpha _\infty ^i,\Lambda ^i,\Lambda '^i,k_0^i,k_0'^i)$ provides the following parameters of the initial state: $\phi ^i=0.84$, $\alpha _\infty ^i=1.23$, $\Lambda ^i=63 ~\mu \text {m}$, $\Lambda '^i=124 ~\mu \text {m}$, $k_0^i=8.52\times 10^{-9}\text { m}^2$, and $k_0'^i=12\times 10^{-9}\text { m}^2$. The corresponding results are plotted (red curves) in Fig. 2c–g and are found in better agreement than those obtained assuming a periodically aligned arrangement of fibers. In particular, the tortuosity value is now in much better agreement, thus implying a complex entanglement of the fiber during the formation of the Aegagropilae fiber network. The most often encountered porosity is 0.86. Therefore, the acoustic simulations of the fully natural 3-dimensional (and of the 2-dimensional man modified) sonic crystal were performed with the acoustic parameters reported on Table 1 and marked by a circle in Fig. 2c–g . These values are quite in accordance with usual values encountered for manufactured fibrous materials such as mineral wool, accounting for the fact that the Aegagropilae fiber diameter is much larger.

**Table 1:** Porous parameters of the considered Aegagropilae fiber network.

Porosity, $\phi$	0.86
Tortuosity, $\alpha _{\infty }$	1.2
Viscous characteristic length, $\Lambda$ ($\mu$m)	73
Thermal characteristic length, $\Lambda '$ ($\mu$m)	145
Static viscous permeability, $k_0$ (10$^{-9}$m$^2$)	1.21
Static thermal permeability, $k_0'$ (10$^{-9}$m$^2$)	1.70

These results apply several comments. The pore constrictions are around $\Lambda \approx 60~\mu \text {m}$, while the pore radius $\Lambda '\approx 120~\mu \text {m}$, which is again consistent with the stereo microscopic image of the fibers Fig. 2b. The ratio between these two lengths is around 2 which is consistent with usual fibrous materials. The fiber arrangement is complex as testified by the large value of the tortuosity. Although the number of samples is not sufficient to develop a probability density function, acoustic characterization seems to be a simple and efficient tool to assess the microstructure of Aegagropilae fiber network.

### Fully natural 3-dimensional sonic crystal

#### Crystal manufacturing and testing

Half of the 15 remaining natural Aegagropilae spheres were cut into four parts, resulting in quarter-spheres. Six Aegagropilae quarter-spheres of radii $20\pm 1 \text { mm}$ are placed in a square cross-sectional impedance tube with side $d\big / 2=21.5\text { mm}$, their centers being separated by a distance $d=43\text { mm}$, see Fig. 1d. Double sided tape was used to ensure the correct boundary condition between the Aegagropilae quarter-spheres and the rigid walls of the impedance tube. As these rigid walls act as perfect mirrors, a perfectly periodic pattern is created in the perpendicular plane to the tube axis, i.e., in the $(x_1,x_2)$ plane as depicted in Fig. 1d. Therefore, a finite depth perfectly periodic structure is created, composed of six layers incorporating spheres arranged over a square lattice in the $(x_1,x_2)$ plane, thus mimicking a finite depth primitive cubic system with a filling fraction $\,f\!\!\!\!\!f\approx 0.4$. The reflection and transmission coefficients are measured via 4 flush mounted microphones for frequencies below the cut-off frequency of the impedance tube, $f_{c}=c_0/d\approx 7900 \text { Hz}$. Under this condition, the impedance tube modes exactly match the Bloch ones for normal incident plane wave. Note the cut-off frequency of the impedance tube exactly matches that of the Wood anomaly^28^, which corresponds to the cut-on frequency of the higher order Bloch waves. It is also that of the second Bragg interference in this case. Effectively, the transmission coefficient is so low for frequencies higher than $\approx 7000 \text { Hz}$, that the results are presented only for frequencies below this value. Nevertheless, the first Bragg interference is captured in the measured frequency range.

#### Scattering properties and dispersion relation

**Figure 3:**
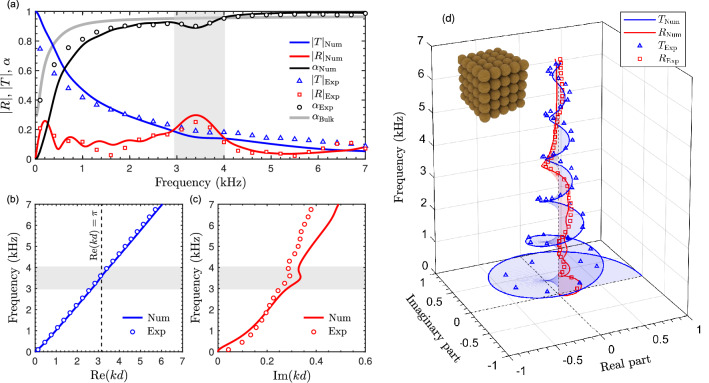
Scattering properties of a 3D primitive cubic arrangement of periodicity $d=43$ mm, made of $N=6$ Aegagropilae spheres of radius 19.5 mm. (**a**) Absorption, reflection and transmission coefficients calculated numerically (black, red and blue continuous curves) and measured experimentally (open black circles, open red squares, and open blue triangles). The grey continuous line represents the absorption coefficient of a bulk material of identical thickness. (**b**) Real and (**c**) imaginary parts of the complex wave number reconstructed numerically (continuous lines) and experimentally (markers). (**d**) Argand diagram of *T* (blue curve) and *R* (red curve) over the frequency range [0 Hz,7000 Hz] obtained numerically (continuous) and experimentally (markers).

Figure 3a depicts the absolute value of the reflection and transmission coefficients, |*R*| and |*T*|, as well as the absorption coefficient $\alpha =1-|R|^2-|T|^2$ measured experimentally and calculated by the Finite Element Method. This numerical model is validated against experiments and multiple scattering theory on the 2-dimensional man modified sonic crsytal in the Supplementary information. Note that |*R*| and |*T*| are different from the reflection and transmission coefficients in energy, i.e., $|R|^2$ and $|T|^2$. Both measured and simulated coefficients are in good agreement assuming sphere radii of $19.5 \text { mm}$ and fiber network properties from Table 1. The discrepancies, notably noticed for the reflection coefficients are attributed to the variability of the radii of the quarter-spheres, of the acoustic properties of the natural fiber network, but also to the presence of the Aegagropilae outward shell. The acoustic influence of this shell is thus weak at these frequencies. The Bragg interference is clearly noticed around $f_{B}\approx c_0/2d\le 3950 \text { Hz}$, as highlighted by the grey regions. While Bragg interference is usually associated with a band gap, preventing the propagation of acoustic wave, therefore leading to a minimum transmission, the weak impedance contrast between the soft Aegagropilae spheres and the air medium almost prevent the band gap translation in the transmission coefficient (see also Supplementary information). Only a smooth drop is revealed in the transmission coefficient. In the opposite, the reflection coefficient presents a peak associated with the Bragg interference, which testifies that destructive/constructive interference occurs. The quasi absence of band gap is also confirmed by the dispersion relation depicted in Fig. 3b,c. The real part of the wavenumber does not present even a change of slope close to the Bragg interference, when $\text {Re}\left( kd \right) =\pi$. Note the value of the first Bragg frequency $f_{B}\approx 3500\text { Hz}$ is lower than that predicted with a value of sound speed of the air medium because sound speed in the fiber network is lower than that in the air. The imaginary part rather presents an inflection point at the location of the Bragg interference. The relative agreement at high frequencies of the imaginary part of the normalized wave vector is mostly attributed to the quarter-spheres diameter variation and the presence of the outward shell. In addition, the reflection coefficient presents five smooth peaks (and six minima) before the Bragg frequency corresponding to the Fabry-Perot interference arising from the six layers (see Fig. 3). These Fabry-Perot interferences are not visible in the transmission coefficient absolute value because of the attenuation.

### Argand diagram and acoustic absorption

To get further insights on the acoustic behavior of this primitive cubic system, the Argand diagram^29^ of *R* and *T*, i.e., their values in the complex plane in function of frequency, are depicted in Fig. 3d. Both *R* and *T* are inscribed in the unitary circle and describe counter-clockwise elliptical loops (with time Fourier convention $e^{-\text {i}\omega t}$) from $R=(0,0)$ and $T=(1,0)$ respectively. While *T* makes half of a loop that encompasses the origin, *R* makes a full loop in the positive real half space in the first bulk band. Each time *R* reaches the appendicular part of the loop towards the origin (*T* also reaches an appendicular part of half a loop) a Fabry-Perot interference occurs. Fabry-Perot interferences are also visible in the Argand diagram of *T*. The attenuation only provides the conic envelop of *R* and *T*, the vertex of which is at high frequency. Of particular interest is that *R* describes half a loop from the positive to the negative real half spaces within the first band gap as a translation of the topological character of the gap^30^. In the present case, *T* still describes a loop around the origin because of the weak band gap translation. However, no Fabry-Perot interference is obviously excited in that band gap because *R* does not move toward the origin. Within the second bulk band, *R* goes back from negative to positive real half spaces around $5500\text { Hz}$ as a manifestation of the symmetry inversion of the system^31^. This band is thus a nontrivial bulk band with two opposite edge states. As a first approximation, the present 3-dimensional system can be effectively assimilated to a 1-dimensional one below the Wood anomaly, when excited at normal incidence. At the edges of each band, $R=r\left( 1-e^{2\text {i} k Nd} \right) \big / \left( 1-r^2 e^{2\text {i} k Nd} \right)$ reduces to the reflection coefficient of an infinite half space *r* thus showing the efficiency of Argand diagram for Zak phase calculation in 1-dimensional system. The most interesting acoustic feature of this primitive cubic system effectively relies on the absorption efficiency. The absorption is higher than 0.8 for frequencies higher than $1\text { kHz}$, although it presents a local minimum at the Bragg frequency due to the associated large reflection. A quasi-perfect absorption peak is also noticed around the symmetry inversion frequency at $5500\text { Hz}$. Fabry-Perot interferences usually coallesce around the frequency of symmetry inversion for two and three-dimensional structures, making possible quasi-perfect absorption by symmetric structures^32^. The absorption coefficient is effectively higher than that of a full $6\times d$-thick layer occupied by the fibrous materials as can be seen in Fig. 3a for most of the frequency range considered.

## Discussion

The acoustic behavior of this fully natural Aegagropilae sonic crystal reveals several outstanding aspects of the fiber network structure and sonic crystals constituted of soft and dissipative medium scatterers, but also links between sonic crystal topology and acoustic properties in the day to day life. First, the fiber network micro-structure has been assessed by acoustical means. The acoustic properties of the Aegagropilae core are found highly correlated to information provided by stereo microscopic image. Acoustic characterization is thus a cheap, easy and efficient tool to study natural fiber entanglement and so Aegagropilae formation. As a corollary, turbulent flow may also represent an excellent sustainable alternative for green manufacturing of non-woven acoustic materials, because Aegagropilae acoustic paramaters were found similar to that of man manufactured acoustic fibrous materials. Interestingly, the outward shell of the Posidonia balls weakly affects the global acoustic behavior of the organic sphere. Second, the dissipative and soft natural sonic crystal was found to exhibit specific fetaures, notably the almost unique translation of the Bragg interference in the reflection coefficient. Nevertheless, the Argand diagram of both *R* ad *T* are found highly informative for the edge state qualification. The second bulk band of this sonic crystal is a nontrivial one with a symmetry inversion. Third, topology analysis of the sonic crystal explains the absorbing efficiency of the structure that largely overcome that of a layer of identical thickness and material. The material gain with respect to this configuration is directly the porosity of a primitive cubic arrangement of the Aegagropilae spheres, i.e., $1-f\!\!\!\!\!f$, and thus is at minimum of $\approx 47 \%$. The absorption coefficient can be further enhanced by increasing the filling fraction (see Supplementary information), which could require considering other type of crystal lattice. For example, cubic body centered or face centered cubic arrangements enable reaching higher $f\!\!\!\!\!f$ than a primitive cubic one and can be more often encoutered in nature. Aegrophilae fiber sonic crystals are also fully natural structures that outperform acoustic properties of usual acoustic material, the use of which does not require transformation. In terms of proving the existence of natural acoustic/elastic metamaterials, Aegagropilae are excellent candidates.

## Methods

### Sample preparation

The 50 samples were dried at room temperature for a month to remove water trapped in the Aegagrophilae. Samples were then placed in a $75 \text { cm}$-depth test sieves with a $710 ~\mu \text {m}$ mesh size and shaken at an amplitude of $1.3\text { mm}$ in a laboratory sieve shaker (CISA BA 200N) for 10 min to remove the sand. Samples were immersed in nitrogen before their cutting to preserve the structural integrity of the fiber network. A hole saw mounted on pillar drills and a ham slicer were used to cut the samples.

### Johnson–Champoux–Allard–Lafarge model

The skeleton of Aegagropilae fiber network is assumed to be motionless and is thus modeled as an equivalent fluid with complex and frequency dependent density and bulk modulus via the Johnson–Champoux–Allard–Lafarge model^23, 24^:

1 $$\begin{aligned} \rho _{eq}=\frac{\rho _0\alpha }{\phi }\text { and }K_{eq}=\frac{\gamma P_0}{\phi }\left( \gamma -\frac{\gamma -1}{\alpha '}\right) ^{-1}, \end{aligned}$$

where $\rho _0$ is the density of the saturating fluid, $\phi$ the open porosity, $P_0$ is the static pressure and $\gamma$ the specific heat ratio. The dynamic and thermal tortuosities, which respectively account for the viscous and thermal losses, read as

2 $$\begin{aligned} \begin{array}{l} \displaystyle \alpha =\alpha _\infty +\frac{\text {i}\nu \phi }{\omega k_0}\sqrt{1-\frac{\text {i} \omega }{\nu }\left( \frac{2 \alpha _\infty k_0}{\phi \Lambda }\right) ^2},\\ \displaystyle \alpha '=1+\frac{\text {i}\nu '\phi }{\omega k_0'}\sqrt{1-\frac{\text {i}\omega }{\nu '}\left( \frac{2 k_0'}{\phi \Lambda '}\right) ^2}, \end{array} \end{aligned}$$

where $\nu =\eta /\rho _0$ is the kinematic viscosity of the saturating fluid, $\eta$ is the dynamic viscosity, $\nu '=\eta /\text {Pr}$ with Pr is the Prandtl number and $\alpha _\infty$, $\Lambda$, $\Lambda '$, $k_0$, $k_0'$ are the tortuosity, viscous and thermal characteristic lengths, and viscous and thermal static permeabilities of the porous medium. In the present case, the surrounding and saturating fluid is air at room temperature, i.e., $\rho _0=1.213\text { kg.m}^{-3}$, $P_0=101325\text { Pa}$, $\gamma =1.4$, $\eta =1.839\times 10^{-5}\text {m.s}^{-1}$, $\text {Pr}=0.71$.

### Experimental set-up

Within the square cross-sectional impedance tube of side $d/2=21.5 \text { mm}$, the acoustic pressures are recorded by 4 microphones, two of which are positioned upstream and the other two downstream of the structure. The sonic crystal depth is $L=6d=258\text { mm}$. The excitation is delivered at one end of the tube by a loudspeaker and takes the form of a sweep sine from $100\text { Hz}$ to $7900 \text { Hz}$ with $\Delta f=10 \text { Hz}$. An anechoic end is placed at the opposite end of the tube. Assuming the anechoic end is not perfect and the structure is reciprocal and symmetric, the scattering pressures are first identified from the four measured pressures, thus enabling the recovery of $R_{\text {Exp}}$ and $T_{\text {Exp}}$ as explained in^22^. The Nicholson-Ross^33^ procedure is then applied to recover the effective wavenumber inside the structure.

### Numerical method

**Figure 4:**
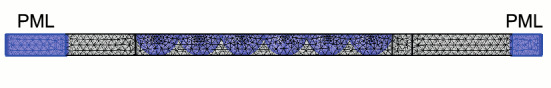
Top view of the numerical domain, exhibiting the tetrahedra elements, Perfectly Matched Layers (PML) locations, and the quarter spheres.

Full wave simulations have been performed using the acoustic module of the Finite Element Software COMSOL Multiphysics 5.2^34^ to numerically reproduce the experimental set-up. The numerical domain is depicted Fig. 4 and is discretized with 14643 tetrahedra elements ensuring that the mesh allows the convergence of the solution in all regions. The model uses the Multifrontal Massively Parallel Sparse direct Solver (MUMPS). The numerical domain geometry is that of the square impedance tube used in the experiments. A background plane wave field excites the system. The two appendicular sides of the numerical domains are covered by Perfectly Matched Layers (PML) to avoid spurious reflections and to numerically reproduce the Sommerfeld conditions (see Fig. 4). The other tube boundaries are rigid ones. At the scatterer boundaries, continuity of pressure and normal velocity is applied, assuming that the scatterer medium is an equivalent fluid represented by the Johnson–Champoux–Allard–Lafarge model. The reflection and transmission coefficients of the analyzed samples (the man modified 2-dimensional and the fully natural 3-dimensonal finite depth sonic crystals) are evaluated from the pressures numerically calculated at the same positions as those of the microphones in the experimental set-up. The Nicholson-Ross^33^ procedure is also applied to recover the effective wavenumber inside the structure. This numerical procedure has been validated against experimental results and Multiple Scattering Theory simulations assuming periodic conditions for the 2-dimensional man modified sonic crystal in the Supplementary information.

## Supplementary information

Supplementary Information

Supplementary Video.
