# Supplementary material for: Natural sonic crystal absorber constituted of seagrass (Posidonia Oceanica) fibrous spheres
Source: Sci Rep. 2021 Jan 12;11:711. doi: 10.1038/s41598-020-79982-9 (PMC7803767; doi:10.1038/s41598-020-79982-9)
Supplement: Supplementary file 1 — Supplementary Information [file 41598_2020_79982_MOESM1_ESM.pdf]

Supplementary information for *Natural sonic crystal absorber constituted of seagrass (Posidonia Oceanica) fibrous spheres*

L. Barguet[1,3,+], V. Romero-García[1], N. Jiménez[2], L. M. Garcia-Raffi[3], V. J. Sánchez-Morcillo[4], and J.-P. Groby[1,+,*]

[1]Laboratoire d'Acoustique de l'Université du Mans (LAUM), UMR CNRS 6613, Institut d'Acoustique - Graduate School (IA-GS), CNRS, Le Mans Université, France
[2]Instituto de Instrumentación para Imagen Molecular, Consejo Superior de Investigaciones Científicas, Universitat Politècnica de València. Camino de vera s/n 46022 València, Spain
[3]Instituto Universitario de Matemática Pura y Aplicada (IUMPA), Universitat Politècnica de València, Camino de vera s/n 46022 València, Spain
[4]Instituto de Investigación para la Gestión Integrada de Zonas Costeras, Universitat Politècnica de València, Paranimf, 46730, Gandia, Spain
[*]To whom correspondence should be addressed. E-mail: Jean-Philippe.Groby@univ-lemans.fr
[+]These authors contributed equally to this work.

Artificial 2D sonic crystal

The acoustic behavior of the 2-dimensional man modified sonic crystal made from Aegagropilae fiber network cylinders, as shown in Fig. 1, is analyzed. The numerical simulation tools, the semi-analytic calculations, and the experimental results are compared. In addition, the Argand diagram is introduced and analyzed to assess the results obtained for this 2-dimensional man modified sonic crystal. Argand diagram was introduced lately as an efficient tool to design acoustic absorbers[1]. This sonic crystal was also considered to validate the numerical procedure used to characterize the fully natural 3-dimensional sonic crystal considered in the main text.

Crystal manufacturing

Six Aegagropilae half cylinders of radii 15 ± 0.5 mm (see Methods) are placed in a square cross-sectional impedance tube of side $d/2 = 21.5$ mm, their centers being separated by a distance $d = 43$ mm and their axis oriented along x_2. Note that the radii of the half-cylinders are slightly larger than that of the saw-hole and those of the samples used for the fiber newtork characterization because they are not maintained. A perfectly periodic structure of finite depth is thus created, similarly to the 3-dimensional fully-natural sonic crystal studied in the main text of the article thanks to the rigid boundaries of the impedance tube. This periodic structure is composed of six layers incorporating infinitely long cylinders arranged periodically along the x_1 axis, thus mimicking a finite depth square lattice system with a filling fraction $ff \approx 0.38$. Below the cut-off frequency of the tube, the impedance tube modes exactly match the Bloch ones for normal incident plane wave.

Scattering properties

Figure 2(a) depicts the refection and transmission coefficients in amplitude and the absorption coefficient as experimentally measured, numerically simulated via Finite Element Method -FEM- (see Section Numerical Modeling of the main text), and calculated via the Multiple Scattering Theory -MST- (see Methods). All curves are in good agreement, with an exact superposition of the FEM and MST simulations. For comparison purpose, the reflection and transmission coefficients in amplitude calculated with the MST in the case of perfectly rigid cylinders are also provided. In this latter case a clear band gap is visible around $f_B = c_0/2d \approx 3950$Hz, with a very low transmission and large reflection due to the Bragg interference. In the lower pass band, the transmission is maximum, i.e., vanishing reflection, at six different frequencies corresponding to the Fabry-Perot interferences occurring within these six layers of periodic cylinders. Of particular interest is the fact that only four peaks in transmission are visible within the second pass-band because of a strong overlapping of two Fabry-Perot interferences. In the soft and lossy case, the transmission coefficient only presents a smooth drop from a frequency band that is slightly lower than f_B, implying the almost absence of Bragg band gap. Only the reflection coefficient presents a peak within this frequency range, thus exhibiting the Bragg interference feature. This is explained by the weak impendance contrast bewteen the air medium and that of the scatterers. In addition, only the reflection coefficient presents drops at the location of the Fabry-Perot interferences. This feature is also testified by the dispersion relations presented in Figure 2(b-c). While the real part of kd

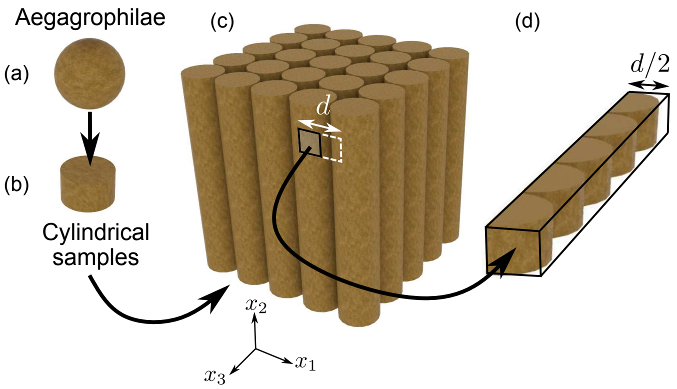

Figure 1. (Color online) (a) Aegagropilae and (b) cutted cylindrical sample used to characterize its acoustic properties. The cylindrical samples were used to construct the 2D man-modified sonic crystal (c) and its corresponding reduced model for the scattering measurement in the impedance tube at normal incidence (d).

vanishes within the band gap, with a large imaginary part in the rigid case, it is almost linear in the soft and lossy case. Only an inflection point is noticed in its imaginary part at the location of the Bragg interference. Please note that the numerically reconstructed wavenumber from the finite depth sonic crystal match that evaluated for the infinite periodic arrangement via MST.

Argand diagram, symmetry inversion and acoustic absorption

The most interesting feature is the absorption encountered in the 2-dimensional man modified sonic crystal, which is higher than 0.8 for frequencies higher than 1000 Hz. At the location of the Bragg interference, it presents a local minimum associated with a maximum of reflection. Of particular interest is the fact that the absorption is quasi-perfect at a frequency within the second pass band. To get a physical insight of this feature, Fig. 2(d) depicts the Argand diagram of R and T, i.e., the value of a quantity in the complex plane in function of the frequency. All elements are located in the unitary circle, because of the energy conservation, and each element describes counter-clockwise ellipical loops, because of the time Fourier convention ($e^{-\mathrm{i}\omega t}$). The results are relatively similar to those of the 3-dimensional fully natural sonic crystal discussed in the main text. The movie FPcoalescence.mp4 depicts reflection, transmission and absorption coefficients and Argand digramm of R and T in the lossy and lossless cases as calculated with MST for scatterer radii varying from 14.5 mm to 15.6 mm. This movie points out the coalescence of Fabry-Perot interferences around the symmetry inversion frequency ($\approx$ 5500 Hz). The lossless case has been calculated by considering only the real parts of the density and bulk modulus used in the lossy case. The coalescence of two Fabry-Perot modes is clearly seen around 5500 Hz, especially on the reflection coefficient in the lossless case. The absorption coefficient at low frequency can be further enhanced by increasing the filing fraction ff. The calculated absorption coefficient of the closely packed 2-dimensional man modified sonic crystal, i.e., when $r = d/2$ thus leading to $ff = \pi/4$, is depicted on Figure 2(a). The absorption coefficient of this closely packed configuration is clearly higher than that of the corresponding bulk material for all frequencies but those around the Bragg interferencies, while less material is used and less volume is occupied.

Methods

Sample preparation

Half of the 15 remining samples was cut in a cylindrical shape with 30 mm in diameter and $\approx$ 21.5 mm in height. These cylindrical samples were then cut in half along their axis, resulting in semi-cylindrical samples.

Multiple Scattering Theory

The Multiple Scattering Theory (MST) is applied to calculate the reflection R_{MST} and transmission T_{MST} coefficients and the acoustic parameters of both 2-dimensional finite and infinite depth structures. The notation adopted in[2] is used. As far as it concerns the finite-depth structures, the upstream and downstream pressure fields read as:

$$
\begin{aligned}
p^u &= e^{\mathrm{i}k_1^i x_1 - \mathrm{i}k_3^i (x_3 - L)} + \sum_{q=-\infty}^{\infty} R_q e^{\mathrm{i}k_{1q}x_1 + \mathrm{i}k_{3q}(x_3 - L)}, \\
p^d &= \sum_{q=-\infty}^{\infty} T_q e^{\mathrm{i}k_{1q}x_1 - \mathrm{i}k_{3q}x_3},
\end{aligned}
\tag{1}
$$

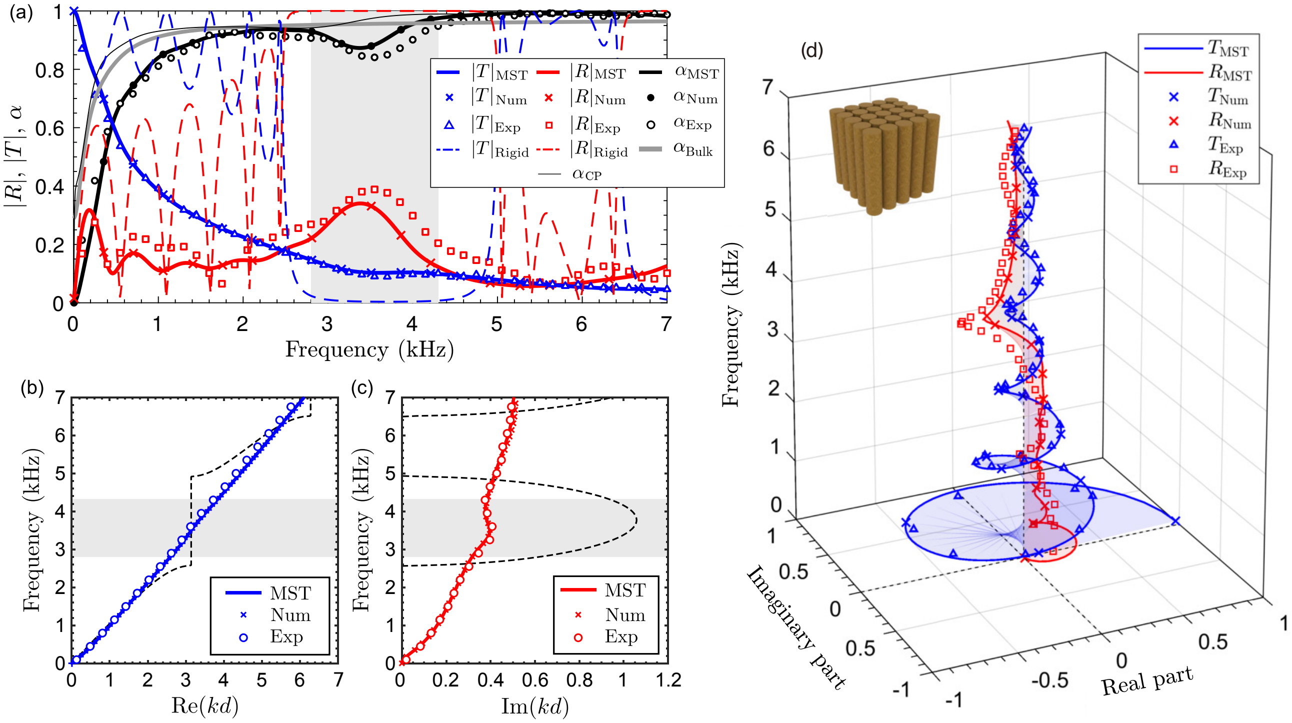

Figure 2. (Color online) Scattering properties of a 2D square periodic arrangement, with periodicity $d = 43$ mm, made of $N = 6$ unit cells of Posidonia cylinders with radius 15.5 mm. (a) Black continuous line (black points) [black open circles] represents the analytical (numerical) [experimental] result for the absorption coefficient. Red continuous line (red crosses) [red open triangles] represents the analytical (numerical) [experimental] result for the reflection coefficient. Blue continuous line (blue x) [blue open squares] represents the analytical (numerical) [experimental] result for the transmission coefficient. Dashed curves represent respectively the transmission (blue) and reflection (red) coefficients in the case of rigid scaterrers. The grey continuous line represents the absorption coefficient of a bulk material of identical thickness. The thin black line represents the absorption coefficient of the corresponding closely packed sonic crystal. (b) Real part and and (c) imaginary part of the complex dispersion relation: continuous lines (crosses) [open symbols] represent the analytical (numerical) [experimental] complex wavenumber. (d) Argand diagram of both the reflection (red curves) and transmission (blue curves): continuous line (open markers) [cross] represents analytical (numerical) [experimental] results.

where $k_{1q} = k_1^i + 2q\pi/d$ and $k_{3q} = \sqrt{k_0^2 - k_{1q}^2}$, with $\mathrm{Re}\left(k_{3q}\right) \geq 0$ are the Bloch waves and R_q and T_q are the reflection and transmission coefficients of the q-th Bloch wave. The incident plane wave is $\mathbf{k}^i = k_1^i \mathbf{x}_1 + k_2^i \mathbf{x}_2 = k_0 \mathbf{x}_2$ at normal incidence. The reflection and transmission coefficients are calculated in the Cartesian coordinate system considering a super-cell comprising 6 identical cylinders via the Multiple Scattering Theory as proposed in[3]. The Schlömilch series are evaluated with the formulae provided by Twersky[4] and the scattering coefficients of a single cylinder takes the form:

$$S_n = \frac{B_n}{A_n} = \frac{\beta_{eq}\dot{\mathrm{J}}_n(\alpha_{eq})\mathrm{J}_n(\alpha_0) - \beta_0\dot{\mathrm{J}}_n(\alpha_0)\mathrm{J}_n(\alpha_{eq})}{\beta_{eq}\dot{\mathrm{H}}_n^{(1)}(\alpha_0)\mathrm{J}_n(\alpha_{eq}) - \beta_0\dot{\mathrm{J}}_n(\alpha_{eq})\mathrm{J}_n(\alpha_0)}, \tag{2}$$

where $\alpha_j = k_j R$, $\beta_j = \alpha_j/\rho_j$, $\mathrm{J}_n(x)$ and $\mathrm{H}_n^{(1)}(x)$ are respectively the first kind Bessel and Hankel functions of order n, and $\dot{\chi(x)} = d\chi(x)/dx$. Below the Wood anomaly, $R_{\rm MST} = R_0$ and $T_{\rm MST} = T_0$.

As far as it concerns the infinite structure, the dispersion relation is determined by solving the following eigenvalue problem

$$\begin{bmatrix} \mathbf{a}_u^+ \\ \mathbf{a}_u^- \end{bmatrix} = \begin{bmatrix} \mathbf{T} & \mathbf{R} \\ \mathbf{0} & \mathrm{Id} \end{bmatrix} \cdot \begin{bmatrix} \mathrm{Id} & \mathbf{0} \\ \mathbf{R} & \mathbf{T} \end{bmatrix}^{-1} \begin{bmatrix} \mathbf{a}_d^+ \\ \mathbf{a}_d^- \end{bmatrix} = e^{\mathrm{i}k_3^B d} \begin{bmatrix} \mathbf{a}_d^+ \\ \mathbf{a}_d^- \end{bmatrix}, \tag{3}$$

where $\mathbf{a}_u^{\pm}$ (resp. $\mathbf{a}_u^{\pm}$) are the complex amplitudes of the waves at the upper (resp. lower) interface of d-thick row of d periodic arrangement of cylinders along the positive and negative x_3 directions, $\mathbf{T}$ is the matrix of components T_q^Q the transmission

coefficient of the q-th Bloch wave when the layer is excited by the Q-th Bloch wave, $\mathbf{R}$ is the matrix of components R_q^Q the reflection coefficient of the q-th Bloch wave when the layer is excited by the Q-th Bloch wave, $\mathbf{0}$ is the zero matrix, Id is the identity matrix, and k_3^B is the normal component of the Bloch wavenumber to be determined. Along the ΓX direction, $k_3^B = k^B$ the Bloch wavenumber.

References

1. Fernández-Marín, A. A., Jiménez, N., Groby, J.-P., Sánchez-Dehesa, J. & Romero-García, V. Aerogel-based metasurfaces for perfect acoustic energy absorption. *Appl. Phys. Lett.* **115**, 061901 (2019).
2. Schwan, L. & Groby, J.-P. *Fundamentals and Applications of Acoustic Metamaterials: From Seismic to Radio Frequency, Volume 1*, chap. 6: Introduction to Multiple Scattering Theory, 143–182 (John Wiley & Sons, 2019).
3. Groby, J.-P., Wirgin, A. & Ogam, E. Acoustic response of a periodic distribution of macroscopic inclusions within a rigid frame porous plate. *Wave Random Complex* **18**, 409–433 (2008).
4. Twersky, V. Elementary function representation of Schlömilch series. *Arch. Rat. Mech. Anal.* **8**, 323–332 (1961).

Additional information

Movie FPcoalescence.mp4 caption: Coalescence of the fourth and third Fabry-Perot interferences in the second nontrivial bulk band associated with symmetry inversion. Reflection and transmission (possibly absorption) coefficients of a 2D square periodic arrangement, with periodicity $d = 43$ mm, made of $N = 6$ unit cells of Posidonia cylinders of radius r ranging from 14.5 mm to 15.6 mm calculated with MST in the presence (upper panel) and in the abscence (lower panel) of viscothermal losses and assocated Argand diagrams. The fourth Fabry-Perot is indicated by the red arraw.
